# Peptidergic control in a fruit crop pest: The spotted-wing drosophila, *Drosophila suzukii*

**DOI:** 10.1371/journal.pone.0188021

**Published:** 2017-11-10

**Authors:** Caroline S. Gough, Grace M. Fairlamb, Petra Bell, Ronald J. Nachman, Neil Audsley, R. Elwyn Isaac

**Affiliations:** 1 School of Biology, Faculty of Biological Sciences, University of Leeds, Leeds, United Kingdom; 2 Insect Control and Cotton Disease Research Unit, Southern Plains Agricultural Research Center, U.S. Department of Agriculture, College Station, TX, United States of America; 3 FERA Science, Sand Hutton, York, United Kingdom; Wake Forest University, UNITED STATES

## Abstract

Neuropeptides play an important role in the regulation of feeding in insects and offer potential targets for the development of new chemicals to control insect pests. A pest that has attracted much recent attention is the highly invasive *Drosophila suzukii*, a polyphagous pest that can cause serious economic damage to soft fruits. Previously we showed by mass spectrometry the presence of the neuropeptide myosuppressin (TDVDHVFLRFamide) in the nerve bundle suggesting that this peptide is involved in regulating the function of the crop, which in adult dipteran insects has important roles in the processing of food, the storage of carbohydrates and the movement of food into the midgut for digestion. In the present study antibodies that recognise the C-terminal RFamide epitope of myosuppressin stain axons in the crop nerve bundle and reveal peptidergic fibres covering the surface of the crop. We also show using an *in* vitro bioassay that the neuropeptide is a potent inhibitor (EC_50_ of 2.3 nM) of crop contractions and that this inhibition is mimicked by the non-peptide myosuppressin agonist, benzethonium chloride (Bztc). Myosuppressin also inhibited the peristaltic contractions of the adult midgut, but was a much weaker agonist (EC_50_ = 5.7 μM). The oral administration of Bztc (5 mM) in a sucrose diet to adult female *D*. *suzukii* over 4 hours resulted in less feeding and longer exposure to dietary Bztc led to early mortality. We therefore suggest that myosuppressin and its cognate receptors are potential targets for disrupting feeding behaviour of adult *D*. *suzukii*.

## Introduction

The spotted-wing drosophila, *Drosophila suzukii*, is a major pest of berry and stone fruit crops that has spread from its native Asia to North America, Europe and more recently South America causing much economic damage [1–5]. Broad spectrum chemical insecticides currently provide the primary control strategy with spinosyns, organophosphates and synthetic pyrethroids being recommended [6–10]. There is however concern over the reliance on these broad spectrum chemicals since insecticide resistance can develop quickly in pest populations which like *D*. *suzukii* have a short generation time and high fecundity [11, 12]. The ever threat of resistance and eventual loss of control using current insecticides provides the impetus to develop new chemical classes with different molecular and physiological targets which would minimise cross-resistance. Insect neuropeptides, their receptors and enzymes involved in peptide metabolism have long been considered as attractive targets for novel chemical control agents (e.g. non-peptide agonists and antagonists) because of their importance in regulating many physiological and developmental processes and the prospect that such targets will deliver species selectivity and fewer environmental problems [13–20]. Neuropeptides are known to regulate insect feeding behaviour, food intake and choice as well as gut physiology, such as the pumping of the crop and peristaltic contractions of the intestine, and therefore disruption of these peptidergic signalling pathways is expected to reduce feeding damage and economic losses from insect pests [21]. The role of neuropeptide signalling in dipteran crop function is of particular interest since this adult organ is important for storing nutrients before passage into the intestinal tract and the malfunctioning of the crop can result in early adult death [22]. In some flies the crop can also function to regurgitate fluid from the mouth, a process that can add moisture to dry food and can also serve to remove excess water from a liquid meal [23]. The movement of fluid into and out of the bi-lobed dipteran crop is determined by a complex system of sphincters and muscular pumps whose activity is modulated by haemolymph serotonin and peptidergic innervation from the hypocerebral ganglion via the crop nerve [23, 24]. Antisera recognising either the RFamide or TDVDHV peptide sequences have been employed to show the presence of a myosuppressin-like peptide (TDVDHVFLRFamide) in the neuronal fibres covering the surface of the crop of the house fly, *Musca domestica*, the blow fly, *Phormia regina* and *Drosophila melanogaster* [25–27]. The myosuppressin sequence (TDVDHVFLRFamide) is highly conserved in dipteran insects and the decapeptide has been shown to be a potent inhibitor of the spontaneous contractions of the crop of *D*. *melanogaster*, *P*. *regina* and *M*. *domestica* [28] [29]. Benzethonium chloride (Bztc), a non-peptidyl mimetic analogue of myosuppressin is myoinhibitory when applied to visceral muscle of several insect species [30–32] and mimics the *in vitro* inhibition by myosuppressin of the spontaneous contractions of the crop of *P*. *regina*, and *M*. *domestica* [26, 27].

Recently, we showed that the amino acid sequences of most of the neuropeptides of *D*. *suzukii* and *D*. *melanogaster* are identical [33] and using mass spectrometry we confirmed the presence of myosuppressin in the nerve crop nerve bundle that innervates the crop muscle. We have now conducted pharmacological studies showing that the spontaneous contractions of the crop are inhibited by both myosuppressin and the agonist Bztc. Furthermore Bztc in the diet suppresses feeding by adult female *D*. *suzukii* and results in early lethality.

## Materials and methods

### Insect rearing

An Italian strain of *D*. *suzukii* was obtained from FERA Science, Sand Hutton, York, U.K. and were cultured in 200 ml plastic bottles with a standard *Drosophila* diet (oatmeal, 7.5%; molasses, 5%; agar, 8.4%; yeast, 8.4%; methyl paraben, 0.35% in water) at 26 ^o^C in a 12h light-12h dark cycle.

### Chemicals

Myosuppressin (TDVDHVFLRFamide) was custom synthesised by Biomatik, Cambridge, Canada. Benzethonium chloride (Bztc) reagent was purchased from Sigma-Aldrich Company Ltd., Gillingham, U.K. Rabbit anti-FMRFamide antiserum was from Peninsula Laboratories, San Carlos, California, U.S.A., whereas the mouse monoclonal GFP Tag antibody (3E6), the Alexa Fluor® 594 goat anti-rabbit IgG and the Alexa Fluor® 488 goat anti-mouse IgG were all from Thermo Fisher Scientific, Paisley, U.K.

### Immunocytochemistry

Tissues were dissected in fly saline [34] and fixed in 4% (wt/v) paraformaldehyde overnight at 4°C. Samples were then washed five times for 2 minutes in 0.3% (v/v) Triton X-100 in phosphate buffered saline (TX-PBS) before incubation with blocking reagent comprising 10% (v/v) goat serum in TX-PBS (GTX-PBS) for 1 hour at 24 ^o^C. Tissue samples were then transferred to the primary antibody (rabbit anti-FMRFamide) diluted 1 in 1000 in 5% GTX-PBS). After 2 days at 4^o^ C, tissues were washed extensively with TX-PBS before incubation for 16 hours at 4^o^ C with the secondary antibody (Alexa Fluor® 594 goat anti-rabbit IgG diluted 1 in 500 in 10% GTX-PBS). Throughout the staining procedure polypropylene tubes containing tissue samples were subjected to a gentle rotation on an orbital shaker. Finally, excess reagent was removed by washing 5 times with TX-PBS (2 minutes for each wash) before the samples were mounted onto microscope slides using Vectashield® mounting medium (Vector Laboratories, Peterborough, U.K.). Slides were stored in the dark at 4 ^o^C until examined using a Zeiss LSM700 Inverted confocal microscope. Epitope specificity was tested by pre-incubating the diluted primary antiserum with 1 μM of myosuppressin peptide for 16 hours at 4^o^ C before using in the same protocol.

### Gut contraction assays

For both the crop and mid-gut contraction assays female D. suzukii, 7–12 days old, were starved overnight with access to water. The following day they were fed a 1M sucrose solution, containing blue food colouring (Supercook ®) for 2 h to highlight the crop. All flies were lightly anesthetised by CO2 and placed onto ice prior to dissection. Saline [34] was added to cover the fly and the abdomen was carefully opened to release the crop and mid-gut. The crop and intact crop duct were carefully dissected and transferred to 45 μL of saline and after a 5 min stabilisation period, the crop contractions were counted visually using a stereoscopic microscope for a period of 1 min. Following this, 45 μL of peptide or Bztc was added using the two pipette transfer system [34] and allowed to stabilise for 1 min. Crop contractions were then counted for 1 min in the presence of the agonist before removal of the agonist by replacement with 45 μL saline. After a 1 min stabilisation period the number of crop contractions were counted for the next 1 min. The same procedure was used to study the effect of peptides and Bztc on peristaltic contractions of the mid-gut except that the gut was not removed from the abdomen, but exposed in 200 μL of saline containing peptide or Bztc. Each data point is the man of five replicates using single tissues.

### Locomotor activity assay

The locomotor activity of adult female *D*. *suzukii* was determined using the Trikinetics DAM2 *Drosophila* activity monitors (Trikinetics Inc. Waltham, MA, U.S.A.) kept in a controlled environment incubator (Memmert GmbH, D-91126 Schwabach, Germany) at constant 25 ^o^C, 60% relative humidity and a 12:12 hour light/dark cycle. Females (7–12 days old) were separated from males under light CO_2_ anesthesia and placed individually in glass tubes (5 x 65 mm) with a plug of agar (2% wt/v) at one end and a ball of cotton wool at the other end. The agar (2% wt/v. of water) contained sucrose (5% wt/v) or sucrose (5% wt/v) plus 5 mM Bztc. To determine the effect of starvation on the activity profile of *D*. *suzukii*, flies were deprived of sucrose by placing them individually in tubes containing agar (2% wt/v of water) only. The tubes were placed in the monitors and the activity was recorded in 1 h time bins. Flies alive after 10 days were transferred to fresh agar/sucrose tubes and data were collected until there was sustained period (12 h) of no activity. The last hour of activity of each fly preceding this 12 h period was recorded. Statistical analysis was conducted using GraphPad Prism 7.01.

### Feeding assay

The effect of Bztc on adult feeding was studied using 5–10 day-old females that, apart from water, had been starved of nutrients overnight before allowing to feed on agar (2% wt/v) made in an aqueous solution of sucrose (5% wt/v) and bromophenol blue (0.5% wt/v). The effect of Bztc on feeding was assessed by including 5 mM Bztc in the sucrose/bromophenol blue solution. Sixty starved flies were used for each of the 3 feeding groups. After 4 h of feeding on the agar, flies were removed and placed in tubes in groups of 5 with access to a capillary tube of sucrose (5% wt/v) for 24 h. The excreted blue faeces were washed from the surface of the tube with 120 μl of water, centrifuged at 13,000 rpm for 10 min before recording the absorbance of the supernatant at 595 nm. Statistical analysis was conducted using GraphPad Prism 7.01.

## Results

### Peptidergic innervation of the crop

**Fig 1:**
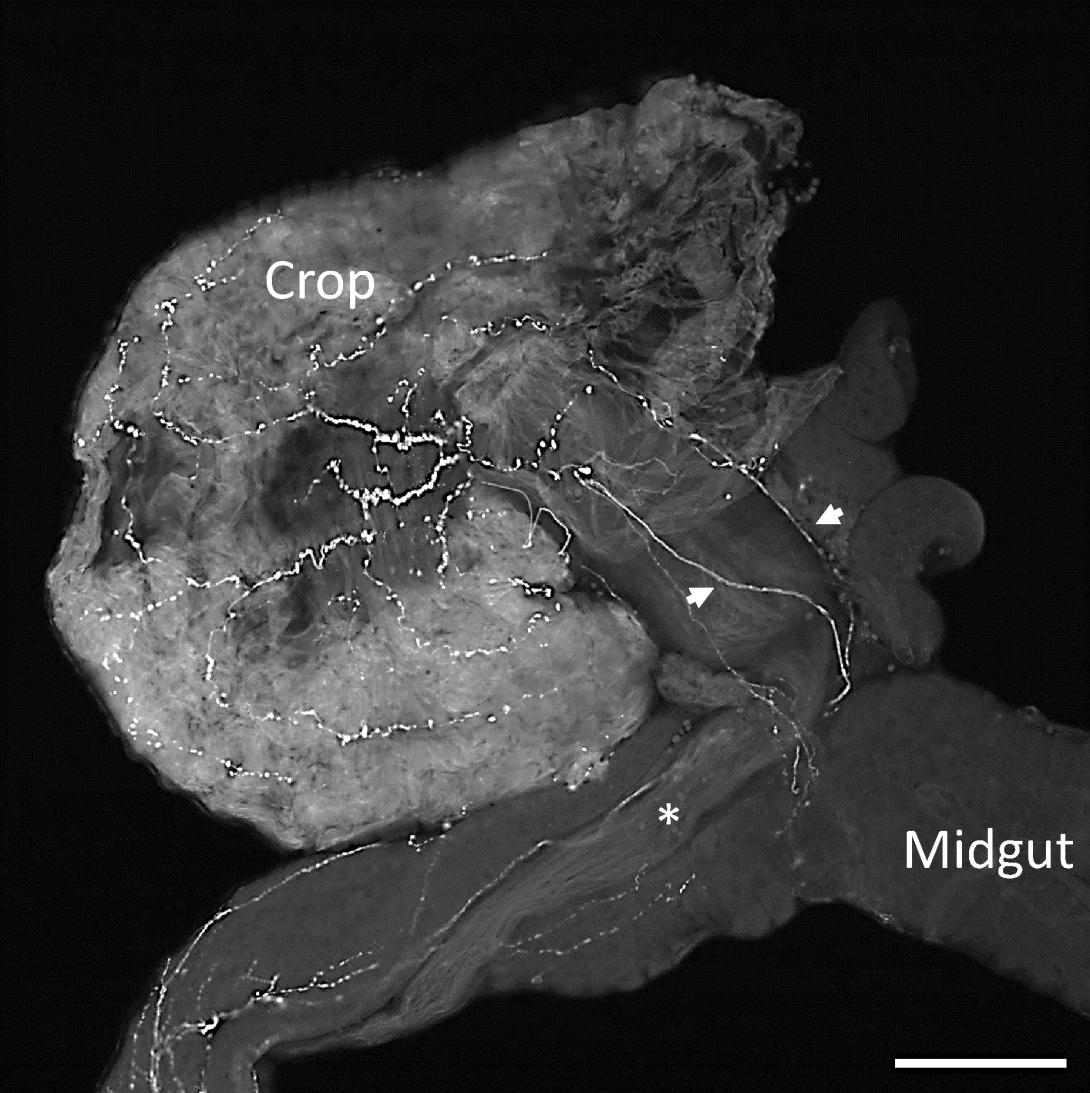
Immunostaining of the crop and crop nerve of *D*. *suzukii* using an antibody recognising the RFamide epitope. Arrows point towards 2 crop nerves that run alongside the crop duct (star) before ramifying across the surface of the crop sac. Scale bar,100 μm.

Antibodies that recognise the C-terminal RFamide of myosuppressin revealed staining of the crop nerve emanating from the hypocerebral ganglion and of ramifying nerve fibres on the surface of the crop itself (Fig 1), consistent with the presence of a peptide with the RFamide sequence at the C-terminus in fibres that are in close proximity with the crop muscle. The RFamide epitope is common to several other *D*. *suzukii* peptides and therefore does not unambiguously reveal the distribution of myosuppressin [33]. The RFamide staining was abolished when the antiserum was pre-incubated with synthetic myosuppressin peptide (S1 Fig)

### Inhibition of crop and midgut contractions by myosuppressin and Bztc

**Fig 2:**
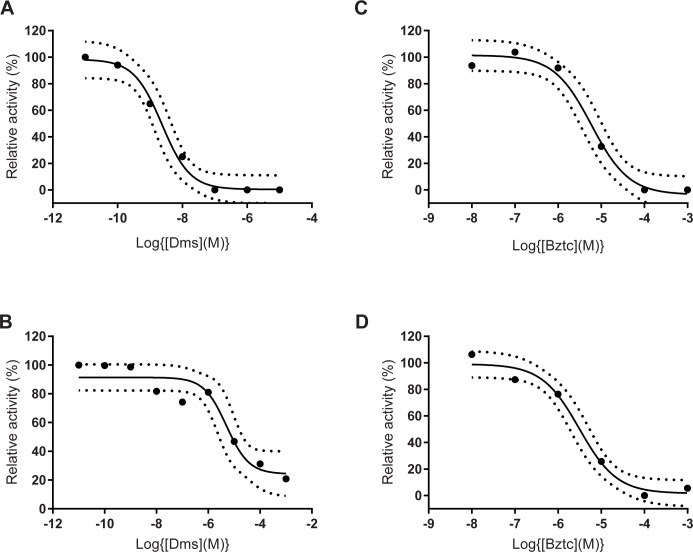
Inhibition of crop and midgut contractions. The effect of myosuppressin (Dms, A and B) and benzethonium chloride (Bztc, C and D) on the spontaneous contractions of the crop (A and C) and midgut (B and D). Data are expressed as the % change in activity (contractions counted in a 1 min period) after the addition of the agonist, as described in the methods section. Values are the mean of 5 determinations using fresh tissues for each determination. Non-linear regression analysis (GraphPad Prism 7.01) was performed to calculate EC_50_ values and to generate the 95% confidence bands.

To determine the pharmacological effect of myosuppressin and the peptide mimetic Bztc on crop muscle activity, we carefully isolated the crop with attached crop duct from adult female flies and counted the spontaneous crop contractions before and after application of these compounds. Myosuppressin was a powerful inhibitor of crop contractions with an EC_50_ of 2.3 nM (Fig 2A). Crop contractions recovered when the peptide was removed from the tissue by washing with fresh saline. The myosuppressin agonist, Bztc, also reversibly inhibited crop activity, but at a much higher concentration (EC_50_ = 5.9 μM; Fig 2C). At this dose the inhibition was reversible, but higher concentrations of Bztc resulted in irreversible inhibition. The effect of myosuppressin and Bztc on midgut peristalsis was also determined and both compounds resulted in inhibition of midgut activity. The inhibition by myosuppressin was much weaker than that observed with the crop (EC_50_ = 5.7 μM; Fig 2B). The peptide however failed to completely suppress midgut muscle activity at high doses. In contrast Bztc was able to completely inhibit peristalsis with a potency (EC_50_ = 3.1 μM; Fig 2D) similar to that recorded for the crop. It was difficult to recover the peristalsis after applying Bztc at doses higher than 1 μM.

### Toxicity of Bztc fed to adult *D*. *suzukii*

**Fig 3:**
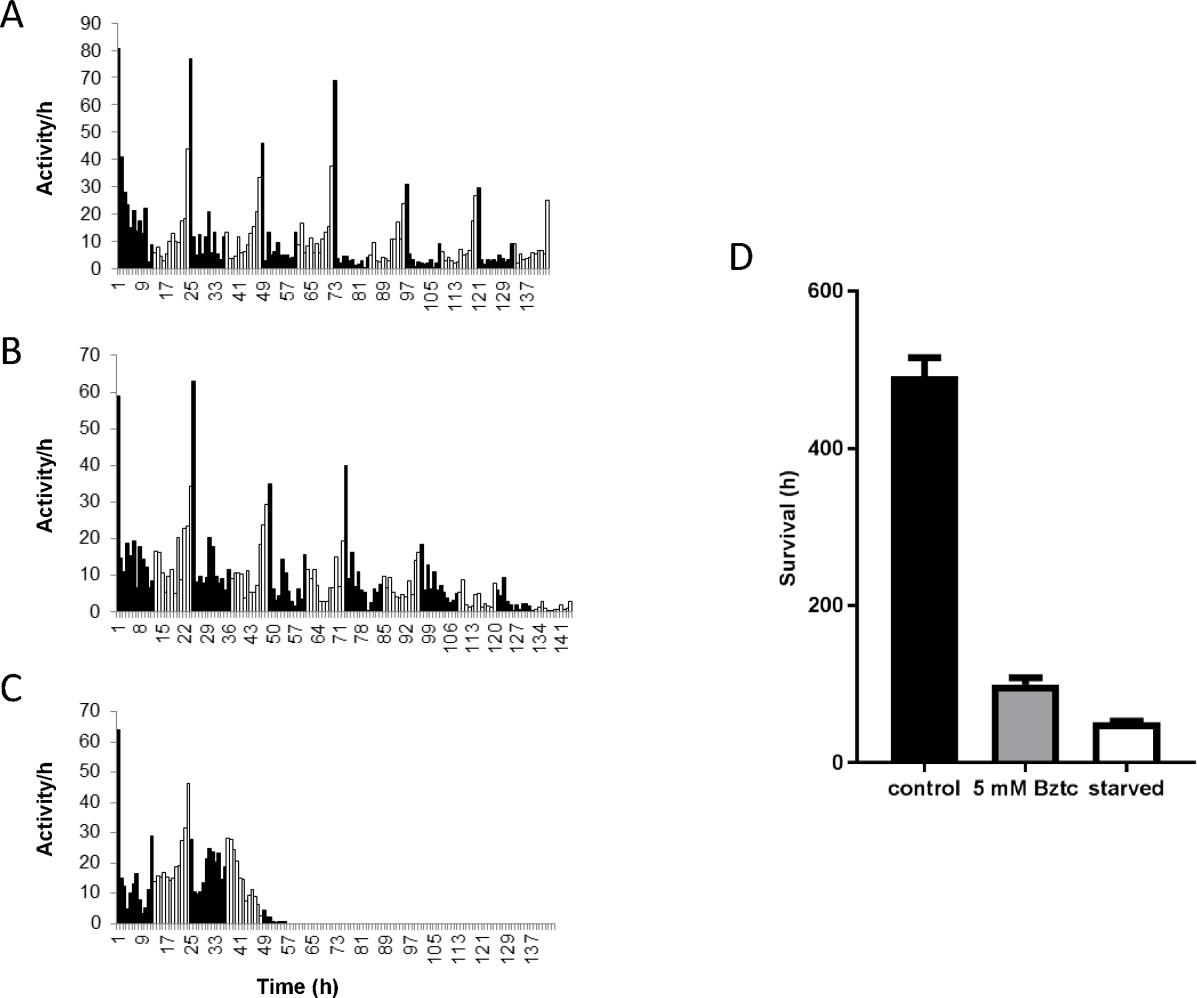
Locomotor activity of adult female *D*. *suzukii*. Flies were maintained in a 12 h:12 h light:dark cycle on (A) sucrose/agar, (B) sucrose/agar with 5 mM Bztc and (C) agar only. Activities for individual flies were recorded using the Trikinetics activity monitors and are expressed as the mean number of beam breaks per hour (n = 32). White and black bars indicate day-time and night-time, respectively. (D) Time of the last recorded activity of the same flies expressed as the mean ± SEM (n = 32). Differences in the mean values are statistically significant (t-test, P <0.001).

The toxic effects of feeding Bztc to adult *D*. *suzukii* was investigated by monitoring the locomotor activity of adult females housed individually in glass tubes with a sucrose/agar plug as an energy source. The experiment was conducted in a controlled environment of constant temperature and humidity and a 12/12 hour light/dark cycle. In the absence of Bztc, flies displayed a rhythmic pattern of daily activity that included a steady rise during the second half of the day leading to a prominent peak of activity at lights-off (Fig 3A). This pattern was maintained for at least 6 days although the total daily activity was reduced after 3 days. The addition of Bztc to the agar/sucrose did not appear to significantly affect the activity profile until day 5 when high mortality occurred (Fig 3B). The time of the last burst of activity for each fly was recorded as a surrogate measure of the time of death. At 5 mM Bztc, this time point was cut from around 500 to 100 h (Fig 3D). Under starvation conditions where only water/agar was provided, flies appeared to succumb around 50 h (Fig 3C & 3D).

### Effect of Bztc on feeding

**Fig 4:**
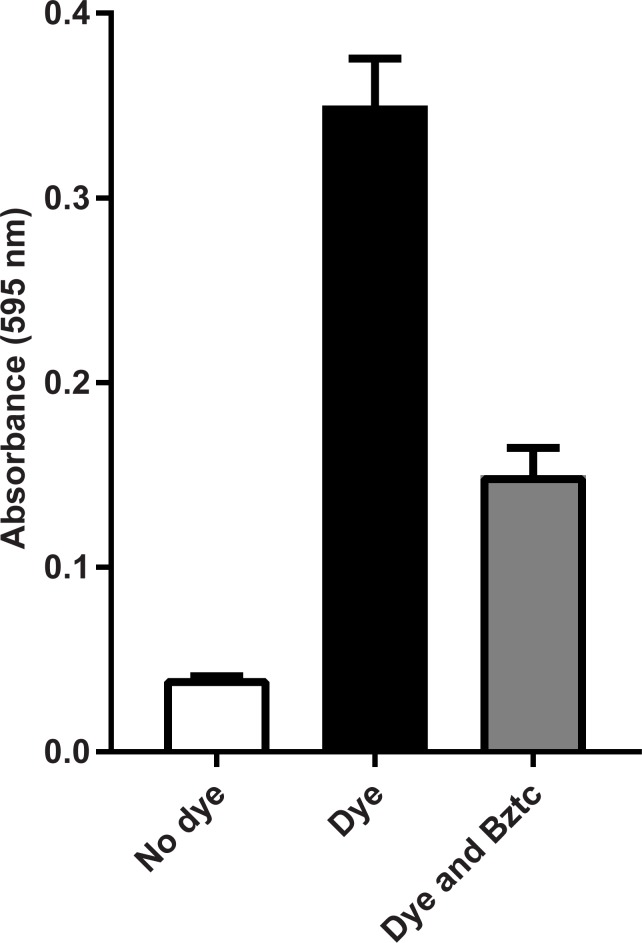
The effect of Bztc on the uptake and excretion of sucrose/food dye by adult female *D*. *suzukii*. The amount of dye in the faeces after 4 h of feeding was determined spectrophotometrically (595 nm) and the results are expressed as the mean ± SEM (n = 12). Differences in the means values are statistically significant (t-test, P <0.001).

Bztc was fed to adult female *D*. *suzukii* in a sucrose diet containing a blue food dye. The amount of dye excreted in the faeces was used as a surrogate assay for food intake over a 4 h period (Fig 4). Bztc reduced the amount of blue dye in the faeces by around 60% suggesting an effect of the chemical on feeding.

## Discussion

Previously we have provided evidence for myosuppressin innervation of the *D*. *suzukii* crop from mass spectrometric analysis of the CNB that provides innervation of the crop from the hypercerebral ganglion [33]. We have now provided additional evidence using an antibody that recognises the C-terminal dipeptide sequence (RFamide) of myosuppressin. Although RFamide is common to other *D*. *suzukii* neuropeptides [33], the presence of myosuppressin in the nerve fibres on the crop surface is expected to generate strong staining with this antibody and is consistent with previous immnocytochemical data generated using a myosuppressin-specific antibody in other dipteran species, including *D*. *melanogaster* [25–27]. The inhibitory action of myosuppressin on the crop of *D*. *suzukii* is also consistent with previous studies on the role of myosuppressin in controlling crop physiology in *D*. *melanogaster* and *P*. *regina*. Two G protein-coupled receptor genes (*DmsR-1* and *DmsR-2*) from *D*. *melanogaster* have been identified as myosuppressin receptors based on their response to nM concentrations of myosuppressin when expressed in mammalian cell lines [26, 35, 36]. The potency of the peptide in the inhibitory action (EC_50_, 2.3 nM) on *D*. *suzukii* crop is somewhat greater than that reported for activating the two *D*. *melanogaster* G protein-coupled receptors (EC_50_, 40 nM) [26, 35, 36] and is consistent with the near 100% inhibition of the contractions of *P*. *regina* crop at a concentration of 25 nM.Myosuppressin also inhibited the peristaltic contractions of the *D*. *suzukii* midgut, but this effect was weaker.

Bztc shares several structural properties with the C- terminal pentapeptide sequence (VFLRFamide) of insect myosuppressins and has been shown to mimic the myoinhibitory actions of these neuropeptides on heart, visceral and skeletal muscle from different insect species. For example Bztc inhibited the spontaneous contractions of the hindgut of the cockroach, *Leucophaea maderae* and suppressed neurotransmitter release from excitatory motor neurons of skeletal muscle of the meal worm, *Tenebrio molitor* [31]. On the *L*. *maderae* hindgut,Bztc inhibits spontaneous contractions at an EC_50_ of 6.4 x 10^−7^ M, comparable with the EC_50_ of 4.2 x 10^−7^ M noted for the C-terminal pentapeptide of cockroach myosuppressin [31]. Bztc also copied the activity of locust myosuppressin by inhibiting proctolin-induced contractions of the oviduct from *Locusta migratoria* in a dose-dependent manner (EC_50_, 6 x 10^−5^ M) [30]. In the same study Bztc competitively displaced radio-labelled myosuppressin from both high and low affinity myosuppressin receptors leading to the conclusion that Bztc was acting as a myosuppresssin receptor agonist in the locust oviduct. Bztc has effects similar to myosuppressin on heart contractions of larvae, pupa and adult stages of *D*. *melanogaster* [32] and the crop of *P*. *regina* [26] and *M*. *domestica* [27]. In the present study we showed that Bztc also inhibits the contractions of both the crop and midgut of adult *D*. *suzukii*, and that it interfered with feeding and greatly shortened life-span when introduced into the fly diet. The potency of Bztc (EC_50_, 3–10 μM) was comparable to that reported for the suppression of locust oviduct contractions (EC_50_, 30 μM) through interaction with the myosuppressin receptor [30]. Although these *D*. *suzukii* data are consistent with Bztc acting as a myosuppressin agonist, it is not certain which receptor or receptors are involved in the signal transduction or whether it is acting through some other mechanism. The FlyAtlas data base reports that the highest expression of both *DmsR-1* and *DmsR-2* amongst adult *D*. *melanogaster* tissues is in the crop [37]. A third receptor gene (*FMRFa-R*, CG2114) from *D*. *melanogaster* can also be activated by nM concentrations of myosuppressin [38, 39], but this gene, unlike *DmsR1* and *DmsR2*, is not expressed in the crop to any significant extent and therefore less likely to have a physiological role [37]. When *DmsR-1* and *DmsR-2* were functionally expressed in mammalian CHO cells, Bztc failed to elicit a specific response which leaves open the possibility that this agonist of locust myosuppressin inhibits *Drosophila* crop muscle through one or more other mechanisms [35]. Indeed the observation that the contractions of both the crop and midgut could not be recovered in our assay protocol when the final Bztc concentration was above 10 μM might suggest non-specific effects at the higher concentrations, possibly due to the weak surfactant properties of this quaternary ammonium salt [40].

Peptides such as myosuppressin are unlikely to survive for long in the intestine of insects and therefore the design of more stable peptide mimetics or completely non-peptide agonists or antagonists, such as Bztc, is most desirable. The *in vitro* demonstration that Bztc blocks gut contractions and the *in vivo* toxicity of the chemical when administered in the diet provides support for targeting myosuppressin signalling when considering new targets for the development of neuropeptide-based approaches to *D*. *suzukii* control.

## Supporting information

S1 Fig Antibody specificity experiment. Bright field (A) and confocal (B) micrographs of a crop, crop duct and crop nerve bundle stained with an antibody recognising RFamide that had been pre-incubated with 1 μM myosuppressin. There is total lack of neuronal staining when the antiserum is blocked with synthetic peptide.
(TIF)
