# Supplementary figures and images for: Peptidergic control in a fruit crop pest: The spotted-wing drosophila, Drosophila suzukii
Source: PLoS One. 2017 Nov 10;12(11):e0188021. doi: 10.1371/journal.pone.0188021 (PMC5681264; doi:10.1371/journal.pone.0188021)

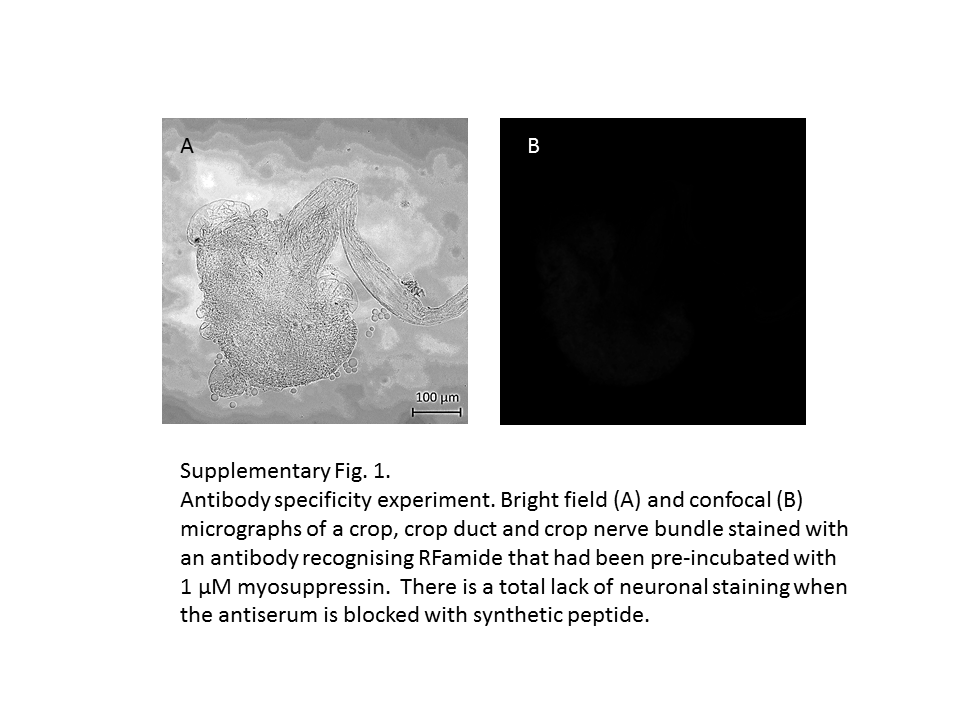

Supplement: S1 Fig — Bright field (A) and confocal (B) micrographs of a crop, crop duct and crop nerve bundle stained with an antibody recognising RFamide that had been pre-incubated with 1 μM myosuppressin. There is total lack of neuronal staining when the antiserum is blocked with synthetic peptide. (TIF) [file pone.0188021.s001.tif]
